# Mammary gland 3D cell culture systems in farm animals

**DOI:** 10.1186/s13567-021-00947-5

**Published:** 2021-06-02

**Authors:** Laurence Finot, Eric Chanat, Frederic Dessauge

**Affiliations:** PEGASE, INRAE, Institut Agro, 35590 Saint Gilles, France

**Keywords:** Mammary gland, Epithelial cell, Organoid, 3D culture

## Abstract

In vivo study of tissue or organ biology in mammals is very complex and progress is slowed by poor accessibility of samples and ethical concerns. Fortunately, however, advances in stem cell identification and culture have made it possible to derive in vitro 3D “tissues” called organoids, these three-dimensional structures partly or fully mimicking the in vivo functioning of organs. The mammary gland produces milk, the source of nutrition for newborn mammals. Milk is synthesized and secreted by the differentiated polarized mammary epithelial cells of the gland. Reconstructing in vitro a mammary-like structure mimicking the functional tissue represents a major challenge in mammary gland biology, especially for farm animals for which specific agronomic questions arise. This would greatly facilitate the study of mammary gland development, milk secretion processes and pathological effects of viral or bacterial infections at the cellular level, all with the objective of improving milk production at the animal level. With this aim, various 3D cell culture models have been developed such as mammospheres and, more recently, efforts to develop organoids in vitro have been considerable. Researchers are now starting to draw inspiration from other fields, such as bioengineering, to generate organoids that would be more physiologically relevant. In this chapter, we will discuss 3D cell culture systems as organoids and their relevance for agronomic research.

## Introduction

**Figure 1:**
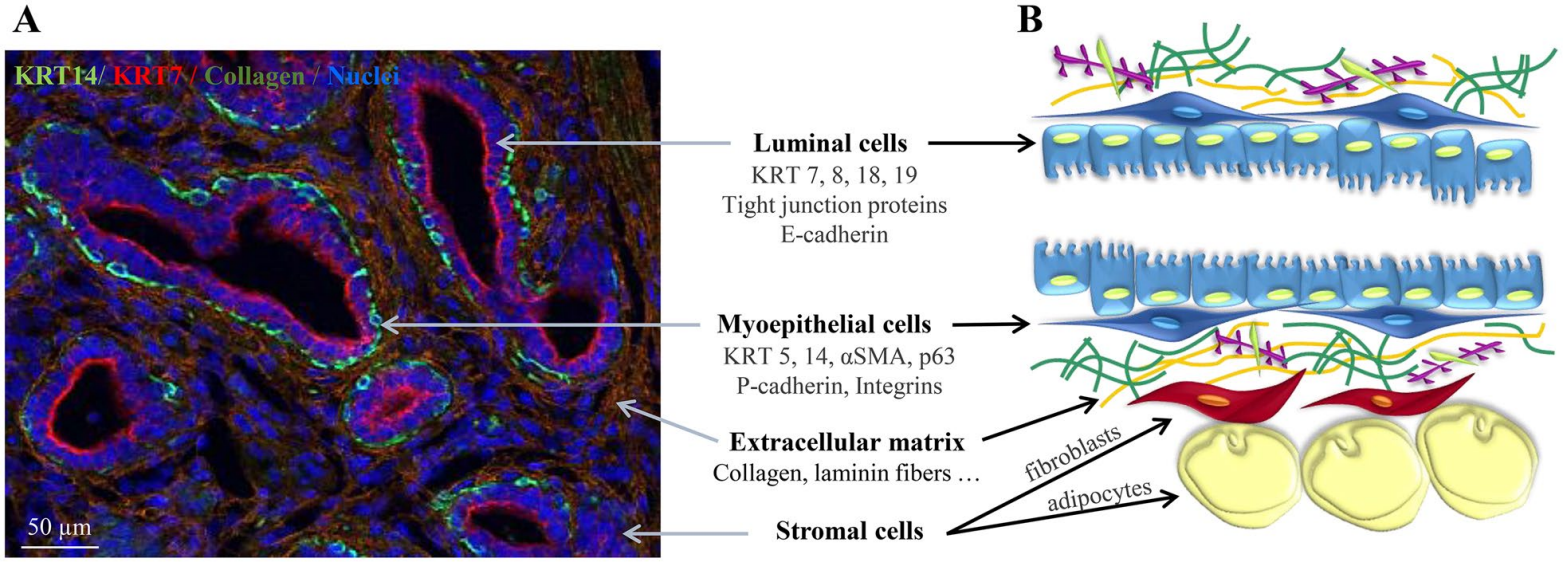
**Organization of the epithelium in lactating cow mammary gland. A** Section of mammary tissue analyzed by indirect immunofluorescence and labeled for the keratins KRT14 and KRT7, and type I collagen for the extracellular matrix. Nuclei were counterstained with DAPI. **B** Schematic representation of the epithelial bilayer with luminal and myoepithelial cells, all separated from stromal cells by the basal lamina constituted of extracellular matrix proteins. The proteins present in each type of cell are indicated in the middle. αSMA smooth muscle actin alpha, KRT keratin. Scale bars = 50 µM.

The mammary gland is structured for optimal synthesis, secretion and ejection of milk products. The secretory tissue, composed of multiple cells assembled into lobes grouped into lobules, is located upstream the nipple (human) or the udder (cattle) relative to the position of both the teat and the gland cistern (in cattle). Lobules are formed of alveoli, or acini, that represent the functional milk production units in the mammary gland. The secretory tissue is drained by a tree network of ducts for the collection of milk which, in ruminants, ends up in the cistern for storage until milking or suckling. The acinus or alveolus consists of a layer of polarized mammary epithelial cells whose apical pole surrounds the alveolar lumen containing the milk they secrete and whose basal pole interacts with contractile myoepithelial cells and the basal laminae. These are embedded within the stromal tissue that contains extracellular matrix proteins, fibroblasts, adipocytes, and lymphatic and blood vessels (Figure 1).

The development of the female mammary gland occurs during defined physiological events related to sexual development and reproduction stages, from fetal life to lactation and beyond [1]. During mammogenesis, from embryogenesis to parturition, mammary cells proliferate and differentiate to set up the different tissues that will form the mammary gland. These cycles of development are driven by the mammary stem cells and their progenitor cells. Cellular and tissue dynamics continue during lactation and between lactations during transitional phases such as dry-off. As a cyclic organ, the mammary gland is subjected to a wide variety of stimuli, mainly hormonal, that condition the remodeling of the tissue during repeated cycles of pregnancy, lactation and involution. In cattle breeds, the adult mammary gland follows successive reproductive/lactation cycles for intensive milk production. For ruminants subjected to breeding schemes promoting optimal lactation, this organ shows a huge ability to adapt to various constraints including breeding practices (nutrition, milking frequency or hormonal treatments) and environmental challenges (climate change) to adjust its level of production while maintaining its health.

Studies of mammary cells biology are of the utmost importance to understand the organization, development and functioning of the acini, and to identify significant clues for the maintenance of mammary gland function and architecture. Cell culture is essential for exploring the processes underlying the development of this organ, such as branching morphogenesis, and the dissection of its biological functions. Our aim here is to provide an overview of the development of three-dimensional (3D) cell culture systems, including current knowledge and advances in the emerging organoid culture systems, for the study of mammary gland biology in farm animals, with a focus on cattle.

## Advances in culturing functional epithelial cells

It is well recognized that two-dimensional (2D) cell culture does not provide optimal conditions to reach the complex organization and the specific cellular contacts observed in the mammary gland in vivo. Indeed, once mammary epithelial cells are removed from their native tissue environment and cultured on standard plastic supports, they lose essential interactions with their microenvironment such as the extracellular matrix (ECM), the other cells of the epithelium including myoepithelial cells, or the stroma. As a direct consequence, their phenotype and morphology change and they lose polarity. For milk-secreting mammary epithelial cells, the lack of relevant polarized morphology results in non-functional differentiation [2]. Most certainly, the signals regulating major cellular functions such as proliferation, metabolism, differentiation and apoptosis are lost following disconnection from the native microenvironment. Although mammary epithelial cells can proliferate as monolayers on plastic, they are subsequently unable to produce key milk proteins such as caseins and to maintain native milk secretory activity. It is only when receiving signals from ECM proteins plus hormones (prolactin, growth factors) that structures similar to those observed in vivo and tissue-specific gene expression (e.g., casein genes) occur [3, 4]. From the 70 s, in vitro mouse mammary culture evolved from basic monolayers of cells to complex 3D culture systems considering the importance of the cell polarization and microenvironment. Three-D and organoids cell culture developments made with murine mammary cells have promptly been adapted to human cells for similar research objectives.

During the development of 3D cell culture systems and organoids, technological advances have been made on several fronts: the nature of the ECMs and culture supports, the co-culture of various cell types and the composition of culture media.

Over the past decades, ex vivo 3D culture assays with murine and human cells have been designed to recapitulate ductal invasion and elongation, morphogenic programs of alveologenesis, as well as functional differentiation [5]. With these aims, several proteins of the extracellular matrix such as collagen or laminin, have been tested to coat plastic dishes as a support sheet for seeding the cells. On collagen gels, cells were shown to reorganize and form 3D structures like those observed in vivo and exhibiting milk protein genes expression [6]. In another rodent model, Streuli et al. demonstrated that mammary epithelial cells grown in laminin-rich gels underwent functional differentiation based on the expression of the casein genes [4]. From this period onward, type I collagen and laminin gels have been widely used in 3D culture systems. However, while cells cultured on collagen gels did indeed form a confluent epithelial layer with surface polarization, they showed low secretory and myoepithelial specializations [7]. To overcome this problem, floating collagen gels have been used to grow primary mouse mammary epithelial cells. Briefly, floating gels consisted in detaching the sheet of collagen matrix with attached cells from the plastic support, making floating culture rafts allowing access to the basolateral surface of the cells. With floating gels, intracellular protein synthesis increased to a constant level through 7 days of culture. These hydrogels, however, did not fit certain specific applications such as in vivo tissue implantation assays due to mass loss after sample transplantation. Composite scaffold gels have therefore been developed to overcome these problems. Addition of mucopolysaccharide hyaluronan to collagen is an example of composite gel created to improve scaffold integrity while reducing mass loss (unpublished results cited [8]). Many natural and synthetic polymeric materials have been investigated as alternatives to ECM proteins for 3D cell culture systems but still remain unsuitable for some specific applications [9].

Nearly 30 years ago, solubilized basement membrane preparation extracted from the Engelbreth-Holm-Swarm (EHS) mouse sarcoma (tumors) was developed as a potent hydrogel for the differentiation of functional mammary epithelial cells, due to its enrichment in specific ECM proteins and factors (laminin, collagen IV, and a number of growth factors). These hydrogels or ECM matrices, now well-known as the commercial mouse-sarcoma derived Matrigel^®^ and Geltrex, helped to model cell and tissue behaviors observed in normal development such as branching morphogenesis. With in vitro technique mixing 3D scaffolds and different culture media, it has even been demonstrated that the cells reform mammary terminal ductal-like structures with well-oriented luminal and basal cells [10, 11]. As example, Nguyen-Ngoc et al., observed epithelial ductal elongation and bifurcation with myoepithelial coverage when seeding mammary organoids of murine epithelial cells in mixture of Matrigel^®^ and collagen gels with growth factors enriched media [10]. Composed of several ECM proteins, Matrigel^®^ appeared to be much closer to the physiological reality of the cellular microenvironment. Unfortunately, its inconsistent composition, especially the concentrations of the growth factors and other biologically active components, confers to Matrigel^®^ a batch-to-batch variability affecting the reproducibility of experiments. Like collagen, Matrigel^®^ is unsuitable for certain experiments. When evaluating the role of mechanical and adhesive inputs on 3D tumor cell growth, invasion, and dissemination, Matrigel^®^ rigidity may limit epithelial growth and impair dissemination. Beck et al. in 2013 demonstrated that tumors preferentially disseminate into collagen I but not into Matrigel^®.^ As for collagen, they successfully tested addition of synthetic polymer mixtures to define the material properties that could induce dissemination into Matrigel^®^ [12]. Some more complex mixtures of stromal and basement membrane proteins exist as Humatrix^®^, a gel containing both basement and non-basement membrane molecules. Due to its composition, this gel exhibits biochemical and biological properties different from those of Matrigel^®^ and was mainly used in human cancer studies.

Advances in biomaterials and tissue engineering science have equipped the biologist with new scaffolds and techniques. The last decade has seen the emergence of innovative technologies based on the combination of 3D matrices and others methodologies, including co-culture systems or use of procedures involving sequential proliferation/differentiation media cultures. Novel 3D cell culture systems incorporating co-culture with stromal cells such as endothelial or fibroblastic cells were developed. Using a 3D fibroblast–epithelium co-culture model in which primary mammary organoids are embedded within Matrigel^®^ matrix together with mammary fibroblasts, Koledova et al. investigated the epithelial-stromal interactions during mammary branching morphogenesis. They obtained primary mammary epithelial organoids and fibroblasts by digesting mammary tissue followed by differential centrifugation. Isolation of fibroblasts from epithelial cells was based on a subsequent selective adhesion procedure. They then co-cultured fibrospheres pre-formed in polyHEMA hydrogel and mammospheres embedded in a mixture of Matrigel^®^ and collagen I. The purpose of these 3D co-culture methodologies is to restore native interactions between epithelial cells and their neighboring stromal cells [13].

More recently, digital design technology was adapted to construct mammary tissue using 3D bioprinting engineering to control the formation of organoids through the “self-assembly” of mammary epithelial cells [14]. This innovative technology was previously defined as “the use of material transfer processes for patterning and assembling biologically relevant materials (molecules, cells, tissues, and biodegradable biomaterials) with a prescribed organization to accomplish one or more biological functions” [15]. It can be considered as a bio-fabrication recapitulating the structure and the function of an organ in miniature. As complex structures mixing living and physical material, the development of these 3D scaffolds faces new challenges, especially in cellular bioprinting in which biological agents are integrated into the material during manufacturing. Efforts concern mainly the problematics of polymers presenting varied properties and viability or the precise control of cell layering dissemination. With human cells, bioprinter also allowed to the establishment of large organoids within mammary-ECM extract derived hydrogels, providing a suitable system for studying epithelial cell interaction with the ECM [16].

The last few years have seen the organ-on-chip technology oncoming, combining bioengineering and microfluidics to mimic the microstructure, the dynamic mechanical properties and the biochemical functionalities of living organs. This technology consists of designing a bionic cellular system on a microfluidic chip possessing the characteristics of the microfluidic technology at a level of miniaturization. It takes the advantage of controlling precisely multiple parameters such as chemical concentration gradients and fluid flows. Mainly used for studies in humans, numerous tissue models including intestine, liver, lung, tumors and muscle have been developed. To our knowledge, however, only mammary tumor cells such as ductal carcinoma cells were studied in this way [17, 18]. Nevertheless, this technology must be mentioned because it might well represent a future for organoids and 3D cell culture of normal mammary gland cells. In one of the rare studies using organ-on-chip, a microdevice consisting of microchannels separated by a semi-permeable membrane mimicking the basement membrane was designed to recreate the 3D structural organization of the human mammary duct. In this device, which was originally developed for drug screening, breast tumor spheroids were co-cultured with human mammary duct epithelial cells and mammary fibroblast cells, in a continuous flow of culture media [17]. Later, a novel microfluidic system established with an in vitro co-culture model of breast cancer cells and human mammary epithelial cells was designed to study the effects of the anti-cancer drugs Paclitaxel and Tamoxifen on tumor migration [19]. Migration of cancer cells (the MDA-MB-231 cell line) was increased after co-culture with human mammary epithelial cells. Similarly, Gioiella et al. used a microfluidic device to simulate breast cancer on a chip and to mimic the epithelial-stroma interactions that occur in the stroma during the invasion of malignant breast epithelial cells [18]. The chip design includes an interface allowing physical contact between the separated stromal and epithelial tumor tissues. This type of co-culture system, which approximates the physiology of the studied organ, makes it possible to investigate biological and cellular mechanisms, as well as the cellular communication between tissues of different origins. Microfluidics is clearly an innovative and promising approach for drug screening. Although the organ-on-chip technology, which is in its infancy, has so far only been implemented in the field of cancerology, this cell culture system best approximates the scale of tissues and organs. It constitutes a relevant avenue for the future of in vitro culture.

In parallel, many efforts have been made to develop medium conditions leading to optimal differentiation of mammary cells and to achieve milk proteins and lipids synthesis. In 1988, Aggeler et al. described a 3D culture procedure including lactogenic hormones (prolactin, insulin and hydrocortisone) able to induce caseins synthesis from primary mouse epithelial cells embedded in floating collagen gels [20]. Lactogenic hormones, especially prolactin, are known to drive epithelial cell differentiation in the mammary gland. The so-called lactogenic culture media are therefore supplemented with variable concentration of prolactin ranging from 3 to 5 µg/mL according to studies. By integrating the key hormone prolactin in their primary organoid culture, Sumbal et al. also obtained a mouse mammary organoid system able to synthesize lipids and caseins, thus modeling the lactation and involution-like processes [21]. Likewise, a differentiation medium complemented with oleic acid, pituitary extract and dexamethasone, was shown to induce the synthesis of major milk constituents such as β-casein, triglycerides and lactose, in a 3D in vitro model of bovine primary mammary epithelial cells grown on a cell culture insert coated with collagen [22]. Hence getting as compliant as possible to the functional structure of the mammary gland remains a challenge to the research in the mammary gland biology and physiology fields.

Compared to murine and human studies, the literature on 3D cell culture systems is scarce for other species, notably farm animals. Obviously, the rodent mammary gland is the most widely studied but both its anatomy and physiology are far from being fully representative of those of the ruminant mammary gland. Moreover, the scientific challenges concerning ruminants substantially differ from those for the murine and human species. Indeed, in these cases, the main interest focuses on milk secretion processes and its upstream counterpart, the development of the mammary gland. In cattle, the investigations rather concern two main science fronts: the exploration of the phases of development of the mammary gland during mammogenesis and the reproduction of the organization and functionality of the mammary cells, both to study the entire lactation cycle, including dry off, and its regulations.

## Development of 3D cell culture in farm animals

Three-D cell culture systems represent pertinent tools for the ex vivo modeling of mammary morphogenesis and organogenesis studies in cattle. Following the development of 3D cell culture systems made from murine and human tissue cells, including organoids culture, these methodologies were later adapted for bovine and other livestock animals. These studies were carried out using bovine primary cells obtained from enzymatically digested mammary gland tissue. A method to isolate lobules of acini from the bovine mammary gland and to further grow acini-derived cells in collagen and floating collagen gels dates from 1982 [23]. In this protocol, fresh mammary tissue was distended by steps of tissue slicing and digestion (mechanically and enzymatically using collagenase and hyaluronidase). Following a long incubation period of gentle digestion (up to 24 h) to lose connective tissue, the released acini lobules were recovered. Grown on collagen and floating collagen gels, the colonies of cells identified as epithelial cells by transmission electron microscopy exhibited few differences regardless of the matrix (floating or non-floating) but the cells stopped increasing in size once the collagen was detached from the plastic support. The authors highlighted the potential of this cell culture system for investigating mitogenic effects of biomolecules (hormones, growth factors,…) on bovine mammary cells by taking account the cell shape and substrate interactions this system permitted [23]. A few years later, bovine primary epithelial cells prepared from lactating mammary tissue grown on floating collagen gel, or not, were used to compare the impact of substratum on cellular morphology (polarization) and secretion of milk protein. When grown on floating collagen gels, cells exhibited a polarized conformation with high differentiation status evidenced by the observation of apical microvilli, tight junctions, and fat droplets surrounded by casein-containing secretory vesicles by transmission electron microscopy. Each milk protein type was found to be differently regulated by substratum detachment, or not, with induction of casein and low level of α-lactalbumin secretion using floating collagen gels [24]. In 1994, Furumura et al. developed an assay that consists in the growth of alveolar-like and ductal-like clumps of cells prepared from mammary tissue collected from pregnant cows of two breeds, Holstein and Angus cows, on collagen coated plates [25]. The enzymatic digestion of the mammary tissue followed by several steps of filtration led to the enrichment of epithelial organoids in the cell suspension. These organoids displayed alveolar-like and ductal-like structures when embedded in collagen. No difference was noticed between Holstein and Angus mammary organoids. This 3D cell culture system allowed to test the role of serum and combined mammogenic hormones on primary mammary cells differentiation [25]. According to this report, the use of collagen gel presented the advantage of mimicking the in vivo conditions and allowed relatively long-term studies.

Many researchers have questioned the influence of the ECM protein components (collagen type IV, entactin, perlecan and laminin) on mammary cell morphology and functioning. With this aim, the impact of the components of the ECM on the maintenance of cell differentiation was compared in a study using mammary epithelial cells isolated from lactating cows [26]. Both the reorganization of functional tight junction using Trans epithelial electrical resistance (TER) and the expression of αS1-casein were evaluated. The authors reported the absence of TER, reflecting a dysfunctional tight junction barrier, in cells grown on Matrigel^®^, in contrast to cells grown on fibronectin or laminin substrata that showed some TER. On the other hand, αS1-casein expression was only detected in cells grown in Matrigel^®^ and fibronectin. However, it would be relevant to look for the other caseins to ensure their lack of expression. Even so, this suggests that in 3D culture, the intrinsic properties of the ECM proteins highly affect the cell differentiation status and the cell-to-cell organization. To date, these approaches were widely used to design mechanistic studies for the analysis of the proliferative and morphogenic potential of bovine primary cells [27] and to examine the impact of the different types of ECM proteins [28].

**Figure 2:**
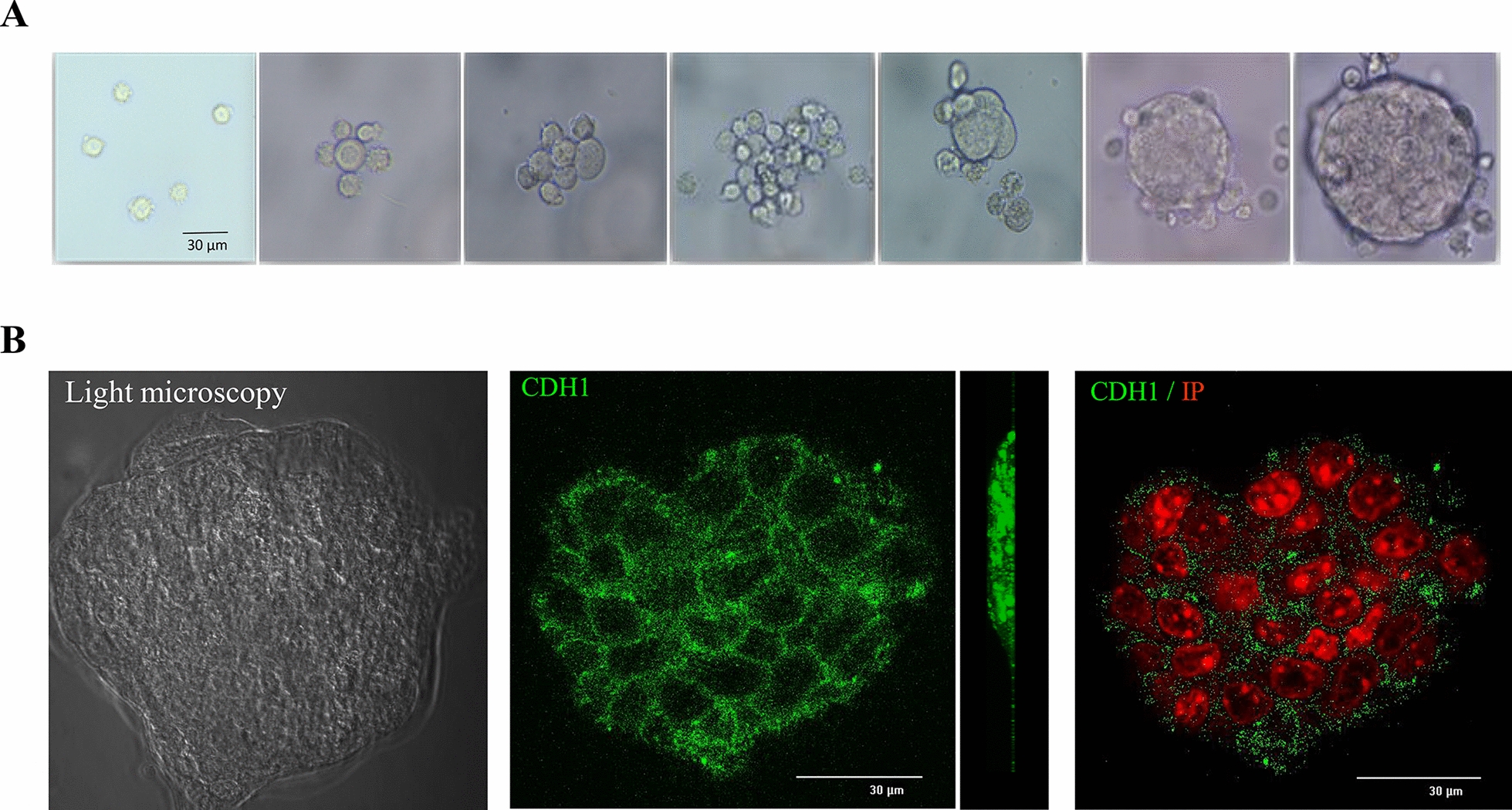
**Three-dimensional cell culture of mammary epithelial cells with Matrigel**^**®**^** gel.** **A** Light microscopy images of mammosphere formation by cell self-assembly (BME-UV1 cell line, ×10 magnification) from the day of seeding to day 9 of culture. **B** Confocal microscopy images of a section of mammosphere in visible light (left) and following indirect immunofluorescence with anti-E-cadherin (CDH1, orthogonal view in the right panel) antibodies and Propidium iodide co-labeling (nuclei staining, CDH1/IP). Scale bars = 30 µM.

In parallel to bovine primary cells, 3D cell culture systems development has also been pursued with bovine mammary cell lines. Kozlowski et al. demonstrated that BME-UV1, a well-known bovine mammary epithelial cell line, formed polarized acinar structures named mammospheres within 16 days when grown with Matrigel^®^. These mammospheres were composed of polarized epithelial cells remaining in direct contact with basement membrane components by recreating the cell-to-cell junction using the standard tight junction proteins zo-1 and e-cadherin, as shown by confocal microscopy. Differential transcriptomic profile analysis between the mammospheres and standard monolayer culture of BME-UV1 allowed for the identification of the genes involved in the regulation of mammary gland epithelial cell polarization and function [29]. Our group has also contributed to the development of 3D cell culture systems based on cell lines (Figure 2). We notably compared the effect of Matrigel^®^ and Ultra low attachment culture support, *vs* standard 2D cell culture, on both the cell phenotypes and morphologies for the two most popular bovine mammary epithelial cell lines, namely MAC-T and BME-UV1 cells. The basal or luminal-like profile of each cell line was characterized and we found that their phenotypes clearly influenced the performances of these cells in 2D or 3D culture. Of note, these experiments provided new information on the lineage of these cell lines and their suitability for 3D culture [30].

Validating a 3D cell culture system in which cells differentiate to synthesize and secrete the constituents of milk, i.e., fully mimicking the secretory activity of acini, remains a challenge. Zhan et al. showed that primary mammary epithelial cells they immortalized from the mammary gland of lactating Chinese cows expressed three of the four caseins (αS1, β and κ) when cultured within Matrigel^®^. Their 3D cell culture system provided appropriate conditions for polarization of the cells as evidenced by the increased expression of the cell-to-cell junction proteins β-catenin and e-cadherin. Interestingly, however, beyond 30 passages of culture, cells embedded in Matrigel^®^ lose their ability to express caseins while maintaining their junctional capacity [31]. On the other hand, bovine primary epithelial cells cultured in a Matrigel^®^ gel were found to express genes involved in milk protein (caseins) and fat biosynthesis but not those for lactose biosynthesis [32]. An elegant study comparing bovine mammary cells cultured on plastic, within collagen or Matrigel^®^ gels, revealed that lactoferrin synthesis, a protein expressed during mammary development, differed with the type of culture substrate considered, the protein accumulating intracellularly when cells were grown within Matrigel^®^ [33]. However, the secreted and intracellular content of lactose and lactoferrin, in addition to β-caseins and triglycerides synthesis, was detected in a 3D culture model of bovine primary mammary epithelial cells cultured on an insert coated with collagen gel. Moreover, the bovine mammary cells grown in this configuration formed continuous and less-permeable tight junctions, assessed by TER, which was accompanied by the establishment of cell polarity [22]. Similar experiments were undertaken using primary mammary epithelial cell cultures established from the mammary tissue of lactating and non-lactating goats [34]. When grown with hormone-supplemented (lactogenic) medium on an ECM-covered (Geltrex) surface, the expression of casein transcripts was detected and increased as compared to 2D cell culture, demonstrating a transcriptional activity of milk protein genes in goat primary mammary cell cultured on ECM gel. In pigs, a species whose lactation is a special issue for the survival of piglets, few studies involving 3D culture of mammary cells have been carried out. Sun et al. have reported the isolation of porcine mammary epithelial cells from lactating sow mammary glands and the expression of milk protein gene transcripts, caseins and ß-lactoglobulin, when cultivated in the presence of a matrix gel [35].

These various studies merely showed that the synthesis of milk constituents requires the polarization of the cells, accompanied by the establishment of cell-to-cell junctions. This was only found when mammary epithelial cells were grown within ECMs. However, the measurement of the activity of secretion due to mammary epithelial cells should not be limited to the expression of certain caseins. Other constituents (lipids, enzymes, lactose…) are as essential. However, efficiency and maintenance of active secretion of milk constituents by mammary epithelial cells of farm animals in 3D culture systems remains to be demonstrated. In conclusion, 3D cell culture system is clearly an important approach for the ex vivo modeling of tissue morphogenesis, organogenesis and biological functioning but, to date, the reconstitution of a fully functional miniature model of the mammary gland has not been achieved for domestic species.

## Reducing animal experiments through organoids culture

To date, very few studies have been conducted with organoids made from mammary gland cells of farm animals due to the delay in the development of such 3D cell culture models. Most of the work, however, was dedicated to the development of organoids from ruminants. Nevertheless, the development of species-specific in vitro models mimicking the detailed mammary gland physiology and function remains an essential alternative avenue for the study of physiological, biochemical, and immunologic functions of cattle mammary cells without resorting to animal experiments with their inherent ethical limits.

An advantage of organoid culture models is that the effect of biomolecules known, or tested, to intervene in key cellular functions can be investigated in vitro at a cellular mechanistic level. For instance, using 3D cell culture of the bovine mammary cell line BME-UV1 embedded in Matrigel^®^, the steroid hormones estradiol and progesterone were not only found to synergically accelerate the development of bovine mammary acini, but also to regulate the genes involved in the induction of the autophagy process occurring in the cells located at the center of the developing spheroids [36]. Using the same 3D culture conditions, Sobolewska et al. investigated the regulation of autophagy by steroid and lactogenic hormones, as well as growth factors during the formation of alveoli-like structures [37]. In the presence of the steroid hormones estradiol and progesterone, acini formation proceeded although autophagy increased. In contrast, the main lactogenic hormone, prolactin, did not affect induction of autophagy within the spheroids. Similarly, the role of serotonin in lactation and involution was investigated using collagen-embedded lactogenic cultures of bovine mammary epithelial cells. Serotonin inhibitors were found to disrupt tight junction assembly as evaluated by TER measurement in 3D cell culture, accompanied by inhibition of milk protein genes expression, highlighting the role of serotonin during the involution process [38]. These studies emphasize the interest of 3D cell culture for exploring autophagic mechanisms at the cellular and tissular levels in order to better understand the involution process, a major issue in bovine. Regarding the investigation of the effects of biomolecules on mammary gland development and function, there are still few in vitro studies but one can predict that their number would probably increase drastically as organoid culture systems will develop further.

Organoid cultures might also help understanding the molecular events occurring during infections targeting the mammary tissue and their dynamic, e.g., during mastitis which is one of the main health problems in the dairy sector. Today, research focuses on better understanding bacterial or viral infection processes that occur in the mammary gland, and the associated immune response, in order to find alternative drugs and treatments to fight the frequent mastitis. Molecules with antimicrobial, anti-inflammatory, immunostimulatory, and antioxidant properties, notably those found in plant, are now considered to prevent or to treat mastitis. One of the research axes concerns the effects of the contact of bacteria with the somatic and epithelial cells of the mammary gland, an interaction which would induce an innate immune response in vivo. This issue can clearly be investigated using organoid culture. It should be mentioned that the main limitation of these cell culture models resides in the absence of immune cells within the system to study the inflammatory response in response to bacterial exposure in situ. To overcome this bottleneck, Le Jan et al. in 2005 developed an organoid culture model reconstructing the caprine mammary tissue, by adding blood immune cells isolated from peripheral blood mononuclear cells of goats to primary mammary cells grown within Matrigel^®^. This 3D cell culture system allowed them to study the local cellular mechanisms regulating the transfer of pathogens (here, the Caprine Arthritis Encephalitis virus) into milk [39]. Combining 3D cell culture and co-culture in such complex culture systems including some cell partners might represent the future for the study of the mechanisms of infection and associated cellular responses, as well as for the development of relevant medical treatments. We must not omit the role of other cell types, such as fibroblasts and adipocytes, in the infection and immune mechanisms and consider integrating them into complex 3D cell culture system.

Another approach to investigate the molecular processes underlying diseases of the bovine mammary gland has been developed by Hillreiner et al. in 2017 whose team sought to establish a 3D cell culture model using primary bovine mammary epithelial cells collected from fresh milk and grown within Matrigel^®^ [40]. The suitability of milk-derived cells for primary cell culture experiments as a functional model of lactation has recently been demonstrated in a comparative assay of milk cells grown on Transwell^®^ vs 3D culture supports [41]. Cells isolated from milk and cultured within ECM gel from EHS murine sarcoma expressed both κ-casein and triglycerides synthesis transcripts and contained intracellular lipid droplets at a higher level than with Transwell^®^ or standard 2D conditions. Obviously, these methodologies present the huge advantage of using a non-invasive source of cellular materials, the mammary epithelial cells exfoliated into milk, thus preserving animal welfare. One disadvantage of this method resides in the fact that a small part of the cells recovered in milk are epithelial cells that have detached from their tissue microenvironment and stood for an undefined time in milk, probably changing their phenotype.

It has to be mentioned that some toxicological tests were carried out using 3D cell culture or organoids making it possible to measure the impact of toxins on mammary tissue. Intoxication by the mycotoxin Aflatoxin B1 is known to be a public health issue and to induce cancer. The cytotoxicity of this mycotoxin and its deleterious effects on bovine mammary epithelial cells during long-term exposure was demonstrated using bovine primary mammary epithelial cells grown in a 3D cell culture system using Matrigel^®^ [42]. Clearly, this approach gives more information on the direct effect of drugs and on the target cells or tissues, as has been shown here for the mammary tissue.

## Conclusion

In ruminants raised to produce milk, understanding the development of the secretory tissue is particularly relevant to apprehend and modulate tissue plasticity in adults during their reproductive cycles. Despite exponentially growing studies using 3D cell culture models in farm animals over the past decade, cattle mammary gland organoids culture models are still in their infancy. Many efforts have been made to adapt the protocols developed with the murine and human species but in vitro research on farm species is still lagging. Major agronomic questions can be tackled with organoid culture, such as testing key biomolecules (xenobiotic, probiotics…) or exploring the dynamics of mammary tissue, and that without resorting to animals. By facing the new innovative challenges to develop 3D cell culture systems, the organoid culture is expected to significantly support the agronomic research on farm animals.

## Abbreviations

2D: Two-dimensional
3D: Three-dimensional
ECM: Extracellular matrix
TER: Trans epithelial electrical resistance
EHS: Engelbreth-Holm-Swarm
